# An Improved Evidential-IOWA Sensor Data Fusion Approach in Fault Diagnosis

**DOI:** 10.3390/s17092143

**Published:** 2017-09-18

**Authors:** Yongchuan Tang, Deyun Zhou, Miaoyan Zhuang, Xueyi Fang, Chunhe Xie

**Affiliations:** School of Electronics and Information, Northwestern Polytechnical University, Xi’an 710072, China; zhuang-my@mail.nwpu.edu.cn (M.Z.); xyfang@nwpu.edu.cn (X.F.); xiechunhe@mail.nwpu.edu.cn (C.X.)

**Keywords:** Dempster–Shafer evidence theory, belief entropy, distance of evidence, IOWA operator, fault diagnosis, sensor data fusion

## Abstract

As an important tool of information fusion, Dempster–Shafer evidence theory is widely applied in handling the uncertain information in fault diagnosis. However, an incorrect result may be obtained if the combined evidence is highly conflicting, which may leads to failure in locating the fault. To deal with the problem, an improved evidential-Induced Ordered Weighted Averaging (IOWA) sensor data fusion approach is proposed in the frame of Dempster–Shafer evidence theory. In the new method, the IOWA operator is used to determine the weight of different sensor data source, while determining the parameter of the IOWA, both the distance of evidence and the belief entropy are taken into consideration. First, based on the global distance of evidence and the global belief entropy, the α value of IOWA is obtained. Simultaneously, a weight vector is given based on the maximum entropy method model. Then, according to IOWA operator, the evidence are modified before applying the Dempster’s combination rule. The proposed method has a better performance in conflict management and fault diagnosis due to the fact that the information volume of each evidence is taken into consideration. A numerical example and a case study in fault diagnosis are presented to show the rationality and efficiency of the proposed method.

## 1. Introduction

The structure of the modern engineering system is more and more complex [1,2], and how to maintain the safety of these systems is a critical problem. Various types of faults may occur because of long-time continuous operation and the changing environmental factors, which may bring great threats to human life [3,4,5,6]. Therefore, fault diagnosis plays an important role in real applications in daily life [7,8,9,10]. In practical applications, a multi-sensor system is widely used in fault diagnosis to make a comprehensive judgment [11,12,13]. For example, fault detection and isolation have been successfully used on the well known Airbus aircraft [14,15], which plays a key role in ensuring the safety of the aircraft [16,17]. However, the information, which may be obtained from a multi-sensor system, is heterogeneous and imprecision [18]. Therefore, it is essential that the uncertain information is pre-processed before data fusion and decision-making [19,20].

Information fusion is a key technology of combining multi-source information [21,22]. To address the uncertain information, some mathematical tools focused on data fusion have been introduced, such as fuzzy sets theory [23,24], Dempster–Shafer evidence theory [25,26,27], comprehensive optimization algorithm [28,29] and so on [30,31,32]. As an important tool in information fusion, Dempster–Shafer evidence theory is widely applied in fault diagnosis [33], pattern recognition [34,35,36,37], multi-criteria decision-making [38,39,40], risk analysis [41,42,43,44], controller design [45,46] and so on [47,48,49]. However, an incorrect result may be obtained if the combined evidence is highly conflicting. To handle this problem, many methods have been presented [50,51,52].

In the frame of Dempster–Shafer evidence theory, while dealing with the conflicting data fusion, one kind of method is to modify the conventional combination rule. Yager modifies Dempster’s combination rule through redistributing the conflicting evidence [53]. However, this method may destroy the good properties of Dempster’s combination rule, such as the commutativity and associativity. In addition, it is unreasonable to blame the combination rule if the incorrect results are caused by sensor failure. Another typical method is to modify the evidence before applying Dempster’s combination rule. Murphy’s method averages the evidence, which does not consider the difference among the evidence [54]. The distance of evidence is used to obtain the weight in Deng et al.’s method [55], which does remedy the disadvantage of Murphy’s method to a certain extent.

In this paper, an improved evidential-Induced Ordered Weighted Averaging (IOWA) sensor data fusion method is proposed in dealing with multi-sensor data fusion in fault diagnosis. Firstly, according to the global distance of evidence $d_{g}$ and the global belief entropy $E_{d}^{g}$, α value of the maximum entropy method (MEM) is established. Namely, the α value is jointly determined by $d_{g}$ and $E_{d}^{g}$. Secondly, a weight vector $W={\left(w_{1},w_{2},\cdots ,w_{n}\right)}^{T}$ is generated based on the MEM model. After that, the evidence are modified by the new IOWA-based weight factor. Finally, the obtained evidence is combined (n−1) times with Dempster’s combination rule. A numerical example and a case study on fault diagnosis verify the validity and reasonability of the proposed method.

This rest of this paper is organized as follows. The preliminaries are introduced in Section 2. In Section 3, a new evidential-IOWA sensor data fusion method is proposed. The application of the new method is presented in Section 4. Conclusions are given in Section 5.

## 2. Preliminaries

### 2.1. Dempster–Shafer Evidence Theory

Dempster–Shafer evidence theory was introduced by Dempster and then developed by Shafer, which is usually applied to manage the conflicting evidence [56,57].

Let Θ be the frame of discernment, and be defined as $\Theta =\{\theta_{1},\theta_{2},\cdots ,\theta_{n}\}$. A basic probability assignment (BPA) $m:2^{\Theta }\to \left[0,1\right]$, is defined as follows [25,26]:

(1) $$\left\{\begin{array}{c} m(\emptyset )=0,\\ \sum_{A\subseteq \Theta }m\left(A\right)=1. \end{array}\right.$$

when m(A)>0, *A* is called a focal element.

Suppose $m_{1}$ and $m_{2}$ are two BPAs on the frame of discernment Θ, Dempster’s combination rule is defined as follows [25]:

(2) $$m\left(A\right)=\left\{\begin{array}{cc} \frac{\sum_{B\cap C=A}m_{1}\left(B\right)m_{2}\left(C\right)}{1-k}, & A\neq \emptyset ,\\ 0, & A=\emptyset , \end{array}\right.$$

where $k=\sum_{B\cap C=}m_{1}\left(B\right)m_{2}\left(C\right)$, is regarded as a measure of conflict between $m_{1}$ and $m_{2}$. The larger the *k*, the larger the degree of conflict.

### 2.2. Jousselme Distance

Jousselme distance is presented to measure of the difference—or the lack of similarity—between any two BPAs, which is introduced as follows.

Let $m_{1}$ and $m_{2}$ be two BPAs on the frame of discernment Θ, then the distance between $m_{1}$ and $m_{2}$ is [58]:

(3) $$d\left(m_{1},m_{2}\right)=\sqrt{\frac{1}{2}{\left(\vec{m_{1}}-\vec{m_{2}}\right)}^{T}\underline{\underline{D}}\left(\vec{m_{1}}-\vec{m_{2}}\right)},$$

where $\underline{\underline{D}}$ is an $2^{\left|\Theta \right|}\times 2^{\left|\Theta \right|}$ matrix whose elements are

(4) $$D\left(A,B\right)=\frac{\left|A\cap B\right|}{\left|A\cup B\right|}\;\;\;\;\;A,B\in 2^{\Theta }.$$

### 2.3. Belief Entropy

Deng entropy is the generalization of Shannon entropy [59], which is defined as follows [60]:

(5) $$E_{d}=-\sum_{i}m\left(B_{i}\right)\log_{2}\frac{m(B_{i})}{2^{|B_{i}|}-1},$$

where $B_{i}$ is a proposition in the BPAs, and $|B_{i}|$ is the cardinality of $B_{i}$.

The entropy can definitely degenerate to the Shannon entropy especially when the belief is only assigned to single element. Namely,

(6) $$E_{d}=-\sum_{i}m\left(C_{i}\right)\log_{2}\frac{m(C_{i})}{2^{\left|C_{i}\right|}-1}=-\sum_{i}m\left(C_{i}\right)\log_{2}m\left(C_{i}\right),$$

and, for $m_{1}\left(A\right)=\frac{2^{\left|A\right|}-1}{\sum_{B\subseteq X}2^{\left|B\right|}-1}$, A,B⊆X, $m_{1}$ is the mass function having the maximum Deng entropy for the frame of discernment X={a,b,c}, and its uncertainty can also be calculated by $\sum_{B\subseteq X}\log_{2}\left(2^{\left|B\right|}-1\right)$.

### 2.4. IOWA Operator

The Induced Ordered Weighted Averaging (IOWA) operator [61], which is introduced by Yager and Filev, is a more general type of the Ordered Weighted Averaging (OWA) operator. An important feature of this operator is that the ordering of the arguments is induced by another variable called the order inducing variable.

Assume there are *n* two-tuple OWA pair $\langle u_{i},a_{i}\rangle ,\;i=1,\cdots ,n$ that has an associated weight vector $W={\left(w_{1},w_{2},\cdots ,w_{n}\right)}^{T}$ of dimension *n* having the following properties:

(7) $$\begin{array}{c} 0\leq w_{j}\leq 1,\\ \sum_{j=1}^{n}w_{j}=1. \end{array}$$

Then, the IOWA operator is defined as follows [61]:

(8) $$F_{w}\left(\langle u_{1},a_{1}\rangle ,\cdots ,\langle u_{i},a_{i}\rangle \right)=\sum_{j=1}^{n}w_{j}b_{j},$$

where $b_{j}$ is the $a_{i}$ of the OWA pair having the *j*th largest $u_{i}$. $u_{i}$ is referred as the order inducing variable and $a_{i}$ is referred as the argument variable.

orness, which is associated with the weight vector $W={\left(w_{1},w_{2},\cdots ,w_{n}\right)}^{T}$, is defined as follows:

(9) $$\alpha =orness\left(W\right)=\frac{1}{n-1}\sum_{j=1}^{n}w_{j}\left(n-j\right),$$

where 0≤orness≤1.

### 2.5. Maximum Entropy Method

To apply the IOWA operator in fault diagnosis, a very crucial issue is to determine its weight. The weight problem is denoted as a constrained nonlinear optimization model in the MEM model, which is presented by O’Hagan. The weight is gained by the following optimization model [62]:

(10) $$\begin{array}{c} Maximize:Disp\left(W\right)=-\sum_{j=1}^{n}w_{j}ln\left(w_{j}\right),\\ S.t\;\;\;orness\left(W\right)=\alpha =\frac{1}{n-1}\sum_{j=1}^{n}w_{j}\left(n-j\right),\\ \sum_{j=1}^{n}w_{j}=1,\\ 0\leq w_{j}\leq 1\;\;for\;j=1,\ldots ,n. \end{array}$$

**Figure 1:**
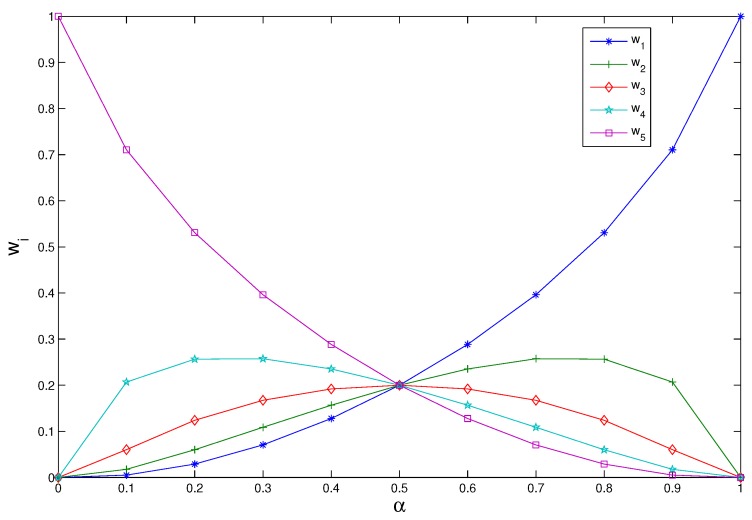
Variation of the weight with orness degree.

Suppose n=5 and the weights satisfy different degrees of orness:α=0,0.1,…,1, then the weight vector is determined by MEM model, which is shown in Figure 1.

From Figure 1, we can conclude that: the value of the weight vector is closer to the average value $W={\left(1/n,1/n,\cdots ,1/n\right)}^{T}$; the value of α is closer to α=0.5; the value of the weight vector is closer to $W={\left(1,0,\cdots ,0\right)}^{T}$; the value of α is closer to α=1. Namely, the smaller the credibility gap among BPAs, the more average for weight distribution.

## 3. The Evidential IOWA-Based Fault Diagnosis Method

**Figure 2:**
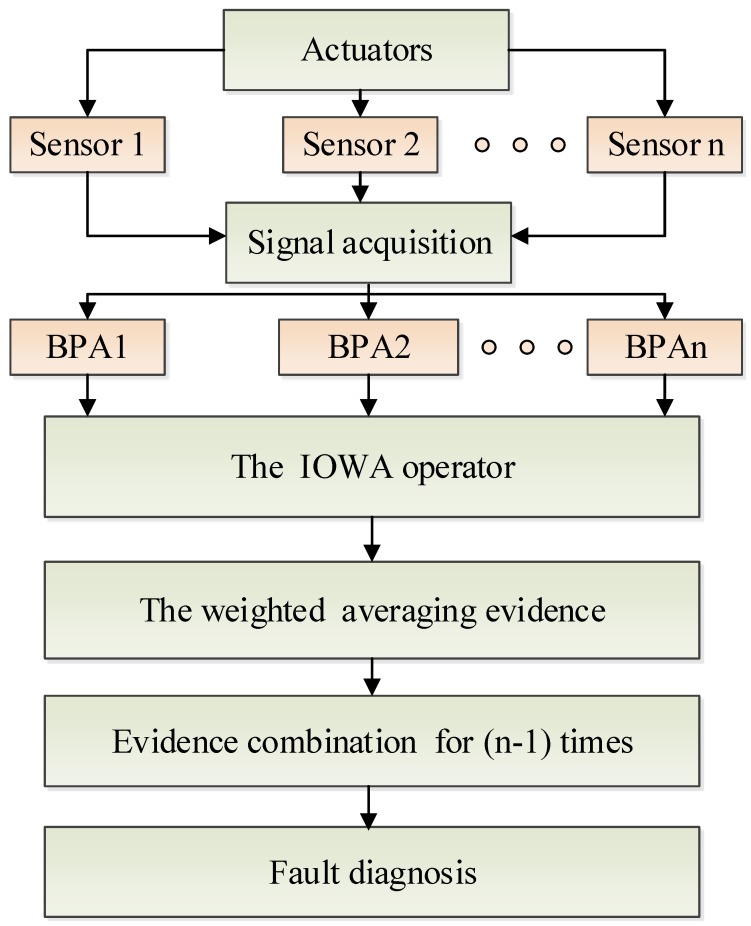
Overall structure of fault diagnosis based on sensor data fusion.

As shown in Figure 2, in the fault diagnosis technique, typically, the first step should be information collecting from actuators. Secondly, all hypotheses are modelled (by BPAs in the frame of Dempster–Shafer evidence theory). Thirdly, the evidence is modified according to the IOWA operator. Finally, data fusion is applied for fault diagnosis and decision-making. Here, how to get an appropriate weight to modify the evidence is very important for locating the possible fault accurately. In the proposed method, the MEM model based on the distance of evidence and the belief entropy are used to generate the appropriate weight of evidence.

### 3.1. The Evidential-IOWA Parameter

Recently, the IOWA operator has aroused the attention of scholars and is widely used in real applications [63,64,65]. However, there are some problems while using the IOWA operator. For example, the α value of a constraint condition usually depends on the experience of the experts, which does not lead to an objective result. In this paper, based on the the distance of evidence and the belief entropy, the α value is induced as an objective weight.

#### 3.1.1. Definition of α in IOWA

The distance of evidence and the belief entropy are jointly considered to determine the α value. The value of α is defined as follows:

(11) $$\alpha =\frac{1}{2}\left(\alpha_{1}+\alpha_{2}\right)=\frac{1}{2}\left(e^{d_{g}\cdot \ln0.5}+0.5^{E_{d}^{g}}\right),$$

where $d_{g}$ is the global distance of evidence, $E_{d}^{g}$ is the global belief entropy, and $0\leq d_{g}\leq 1,\;0\leq E_{d}^{g}\leq 1,\;0.5\leq \alpha \leq 1$. $\alpha_{1}$ is a data-driven value based on the distance of evidence, and $\alpha_{2}$ is another data-driven value based on belief entropy.

#### 3.1.2. Definition of $\alpha_{1}$ Based on the Distance of Evidence

Assume that there are many pieces of evidence for fault diagnosis. The Jousselme distances $d_{ij},\;i,j=1,2,\ldots ,n$ between two evidence $m_{i}$ and $m_{j}$ can be calculated according to Equation (3), and the distance matrix (DM) is defined as follows:

(12) $$DM=\left[d_{ij}\right]=\left[\begin{array}{cccc} d_{11} & d_{12} & \cdots & d_{1n}\\ d_{21} & d_{22} & \cdots & d_{2n}\\ \vdots & \vdots & \vdots & \vdots \\ d_{n1} & d_{n2} & \cdots & d_{nn} \end{array}\right].$$

The average distance of evidence of $m_{i},\;i=1,2,\cdots ,n$, with respect to the other evidence, denoted as $\bar{d_{i}}$, is defined as follows:

(13) $${\bar{d}}_{i}=\frac{\sum_{i=1,i\neq j}^{n}d_{ij}}{n-1},\;\;\;i=1,2,\cdots ,n,$$

then, the global distance of evidence among all the evidence $d_{g}$ is defined as follows:

(14) $$d_{g}=\frac{\sum_{i=1}^{n}{\bar{d}}_{i}}{n},\;\;\;i=1,2,\cdots ,n.$$

If the global distance of evidence $d_{g}$ has a big value, the smaller the global similarity degree among the diagnosed results, the smaller the credibility degree of each sensor. In other words, the smaller the weight gap among the BPAs, the more average the weight distribution is, which means that the value of α is closer to α=0.5. If $d_{g}=1$, which means that the diagnosed fault type of multi-sensor is entirely different; in this case, the credibility degree of each evidence is the same with each other. Thus, the evidence should be assigned the same weight, namely, the weight vector is $W={\left(1/n,1/n,\cdots ,1/n\right)}^{T}$ and α=0.5.

Conversely, the smaller the value of $d_{g}$, the greater the global similarity degree of the diagnosed results, so the BPAs can be represented approximately by less or even one BPA with a high credibility degree. That is to say, the BPA with high credibility degree is given a greater weight and the BPA with a low credibility degree is given a small weight. Thus, the smaller the value of $d_{g}$, the more inequality of the weight distribution, which means the value of α is closer to α=1. If $d_{g}=0$, which means that the diagnosed results are similar, so the BPA can be represented by any BPAs. Considering the consistency of the algorithm, the initial weight is assigned as $W={\left(1,0,\cdots ,0\right)}^{T}$ and α=1.

Based on the above analysis, a relational formula of the degree of orness $\alpha_{1}$ is defined as follows:

(15) $$\alpha_{1}=e^{d_{g}\cdot \ln0.5},$$

where $d_{g}$ is the global distance of evidence, and $0\leq d_{g}\leq 1,\;0.5\leq \alpha_{1}\leq 1$.

#### 3.1.3. Definition of $\alpha_{2}$ Based on the Belief Entropy

Deng entropy is an efficient tool to measure uncertainty, not only under the situation where the uncertainty is represented by a probability distribution, but also under the situation where the uncertainty is represented by the BPAs. Thus, this entropy is used to determine the α value.

The global belief entropy $E_{d}^{g}$ is defined as follows:

(16) $$E_{d}^{g}=\frac{\sum_{i=1}^{n}{E_{d}}_{i}}{n\cdot {\left(E_{d}\right)}_{\max}},$$

where ${E_{d}}_{i}$ is the belief entropy of the evidence $m_{i}$. ${\left(E_{d}\right)}_{\max}$ is the maximum belief entropy on the frame of discernment *X*, which is defined as:

(17) $${\left(E_{d}\right)}_{\max}=\log\sum_{B\subseteq X}\left(2^{\left|B\right|}-1\right).$$

The greater the global belief entropy $E_{d}^{g}$, the greater the global uncertainty of the diagnosed faults. Therefore, the weight distribution should be more average, and the α value is more close to 0.5. If $E_{d}^{g}=1$, it shows that the diagnosed faults is entirely uncertainty, so they should be assigned to the same weight, that is, α=0.5.

The smaller the global belief entropy $E_{d}^{g}$, the smaller the global uncertainty of the diagnosed faults. Then, the BPA can be represented approximately by a few or even one BPA of relatively small uncertainty. Therefore, the smaller the $E_{d}^{g}$, the more inequality the weight distribution, the closer α=1. If $E_{d}^{g}=0$, the BPA can be represented by any BPAs, that is to say, the weight vector is $W={\left(1,0,\cdots ,0\right)}^{T}$ and α=1.

Based on the above analysis, a relational formula of the degree of orness $\alpha_{2}$ is defined as follows:

(18) $$\alpha_{2}=0.5^{E_{d}^{g}},$$

where $E_{d}^{g}$ is the global belief entropy, and $0\leq E_{d}^{g}\leq 1,\;0.5\leq \alpha_{2}\leq 1$.

#### 3.1.4. The Weight Vector of IOWA

After obtaining the parameters α, the weight vector $W={\left(w_{1},w_{2},\cdots ,w_{n}\right)}^{T}$ can be obtained according to the MEM model. Assume that there are *n* BPAs $m_{i},\;i=1,2,\ldots ,n$, the weight vector $W={\left(w_{1},w_{2},\cdots ,w_{n}\right)}^{T}$ can be calculated according to the following steps:

**Step 1**  According to Equations (14) and (15), the global distance of evidence $d_{g}$ and the $\alpha_{1}$ value can be calculated, respectively.
**Step 2**  The global belief entropy $E_{d}^{g}$ and the $\alpha_{2}$ value are obtained by Equations (16) and (18), respectively.
**Step 3**  The α value and the weight vector *W* are calculated based on Equations (11) and (10), respectively.

### 3.2. Multi-Evidential Fusion Model

After getting an appropriate weight vector, the evidence can be modified before using Dempster’s combination rule. The evidence are reordered according to the IOWA operator. Assume there are *n* BPAs, denoted as $m_{i},\;i=1,2,\ldots ,n$, the steps of ordering and evidence fusion are defined as follows:

**Step 1**  Construct the inducing variable $S_{i}$:
  (19) $$S_{i}=1-\bar{d_{i}},\;i=1,\;2,\cdots ,\;n,$$
  where $\bar{d_{i}}$ is the average distance of evidence obtained by Equation (13).
**Step 2**  Obtain the OWA pairs $\langle S_{i},\;M_{i}\rangle ,\;i=1,\;2,\cdots ,\;n$, where $M_{i}$ is the argument variable, namely, it is the BPAs of the evidence $m_{i}$.
**Step 3**  According to Equation (8), the weighted average evidence can be calculated.
**Step 4**  Combine the new evidence with Dempster’s combination rule by (n−1) times.

**Figure 3:**
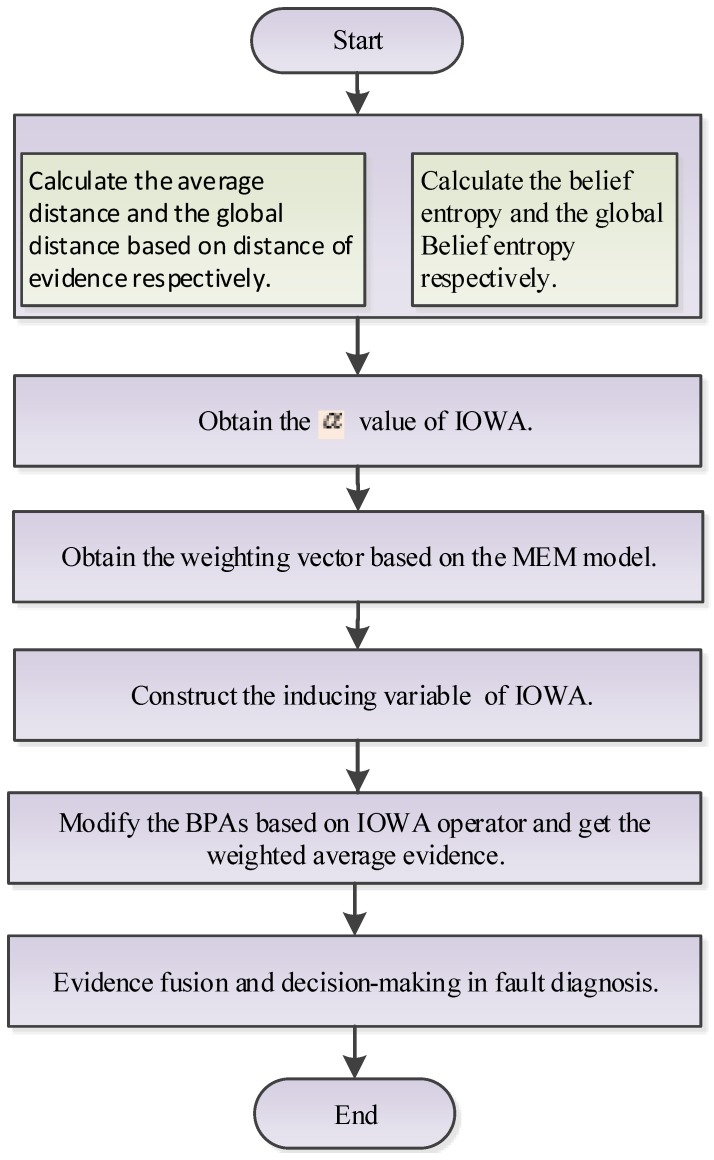
The evidential Induced Ordered Weighted Averaging (IOWA)-based fault diagnosis method.

With the fusion results, decision-making can be made based on the maximum principle of BPAs. An illustrative explanation of the new method is presented in Figure 3. Firstly, the degree of orness α should be computed based on distance of evidence and belief entropy. Secondly, the weight vector $W={\left(w_{1},w_{2},\cdots ,w_{n}\right)}^{T}$ can be obtained based on the MEM model. Thirdly, a corresponding inducing variable can be constructed. Fourthly, evidence modification and fusion can be achieved. Finally, decision-making in fault diagnosis is based on the fused results.

## 4. Application

### 4.1. Experiment with Artificial Data

This numerical example is used to illustrate how to apply the proposed method in fault diagnosis. Assume that, in the case of motor rotor fault diagnosis, vibration signal is collected by five sensors. There are three faults, denoted as *A*, *B* and *C*, in motor rotor, which represents the unbalance, misalignment and pedestal looseness fault types, respectively. The BPAs based on these sensors are assumed to be independent and there are abnormal sensor reports, as is shown in Table 1. Intuitively, $m_{2}$ comes from abnormal sensor report. Since evidence modelling is another open issue in Dempster–Shafer evidence theory, we do not discuss how to model data with BPAs in this paper. For more detail on how to generate BPAs, please refer to some related work such as [45,46,49].

According to the new method shown in Figure 3, firstly, with Equations (13) and (14), the average distance of evidence $\bar{d_{i}},\;i=1,2,\ldots ,5$ and the global distance of evidence $d_{g}$ can be calculated, respectively, and the results are: $\bar{d_{1}}=0.3456,\;\;\;\bar{d_{2}}=0.6647,\;\;\;\bar{d_{3}}=0.2661,\;\;\;\bar{d_{4}}=0.2564,\;\;\;\bar{d_{5}}=0.2641$ and $d_{g}=0.3594$. With Equations (5) and (16), the belief entropy $E_{di},\;i=1,2,\ldots ,5$ and the global belief entropy $E_{d}^{g}$ can be calculated, respectively, and the results are: $E_{d1}=1.5664,\;E_{d2}=0.4690,\;E_{d3}=1.8092,\;E_{d4}=1.8914,\;E_{d5}=1.7710$ and $E_{d}^{g}=0.3534$.

Secondly, the degree of orness α can be calculated by Equation (11):

(20) $$a=\frac{1}{2}\left(e^{0.3594\cdot \ln0.5}+0.5^{0.3534}\right)=0.7811.$$

The weight vector $W={\left(w_{1},w_{2},w_{3},w_{4},w_{5}\right)}^{T}$ is calculated according to Equation (10), and the result is:

(21) $$W={\left(0.5026,\;0.2592,\;0.1337,\;0.0689,\;0.0356\right)}^{T}.$$

In addition, the inducing variable $S_{i},\;i=1,2,\ldots ,5$ are calculated according to Equation (19):

(22) $$\begin{array}{c} S_{1}=1-0.3456=0.6544,\\ S_{2}=1-0.6647=0.3353,\\ S_{3}=1-0.2661=0.7339,\\ S_{4}=1-0.2564=0.7436,\\ S_{5}=1-0.2641=0.7359. \end{array}$$

Thirdly, according to the ordering variable $\bar{d_{i}}$, the OWA pair $<S_{i},\;M_{i}>,\;\;i=1,\;2,\cdots ,\;5$ are ordered as follows:

(23) $$\begin{array}{c} \langle 0.7436,M_{4}\rangle ,\\ \langle 0.7359,M_{5}\rangle ,\\ \langle 0.7339,M_{3}\rangle ,\\ \langle 0.6544,M_{1}\rangle ,\\ \langle 0.3353,M_{2}\rangle . \end{array}$$

Then, the BPAs on each fault are modified according to Equation(8), and the weighted average evidence is:

(24) m(A)=0.5411,m(B)=0.1325,m(C)=0.0217,m(AC)=0.3047.

Finally, combining the weighted average evidence with Dempster’s combination rule by four times, the final results are shown as follows:

(25) m(A)=0.9914,m(B)=0.0001,m(C)=0.0025,m(AC)=0.0061.

In Table 2, we compare the results among several existing methods. It also shows the process of locating the fault type. With the new method, the belief in the fault diagnosis results that *A* is the fault type is 99.14%, which is not lower than the other methods.

In addition, if Dempster’s combination rule is used directly, due to the conflicting evidence $m_{2}$, incorrect results are obtained. The same diagnosis results can be obtained according to the Murphy’s method [54], Deng et al.’s method [55] and the proposed method. However, Murphy’s method is only a simple arithmetic mean which does not consider the difference among the evidence, while Deng et al.’s method ignores the influence of evidence itself in generating the weight factor. The proposed method takes into consideration more available information before making data fusion and fault diagnosis, e.g., the distance of evidence and the belief entropy.

### 4.2. A Case Study

In order to verify the effectiveness and success of the proposed evidential-IOWA sensor data fusion approach, the new method is applied to a case study adopted from [66].

Recall the fault diagnosis problem in [66]. Three potential fault types are denoted as $F_{1}$, $F_{2}$ and $F_{3}$; thus, the fault hypothesis set is $\Theta =\left\{F_{1},F_{2},F_{3}\right\}$. Three sensors report the diagnosis results independently, the diagnosis results are modelled as three bodies of evidence, denoted as $E_{1}$, $E_{2}$ and $E_{3}$, and the BPAs of the diagnosis results are shown in Table 3. Intuitively, $F_{1}$ is the fault type because both $E_{1}$ and $E_{3}$ have a belief of more than 60% on the fault type $F_{1}$, while the $E_{2}$ may come from an abnormal sensor in comparison with the other two bodies of evidence. This is a challenge for data fusion, especially for some conventional combination rules, such as Dempster’s rule of combination. The proposed method is applied to solve this problem.

According to the proposed method shown in Figure 3, the first step is to calculate the average distance and global distance of the evidence $E_{1}$, $E_{2}$ and $E_{3}$. Based on Equations (13) and (14), the calculation results of the average distance of each piece of evidence, denoted as $\bar{d_{i}}\left(E_{i}\right)$, i=1,2,3, and the global distance, denoted as $d_{g}\left(E_{i}\right)$, i=1,2,3, are shown in Table 4.

Then, based on Equations (5) and (16), the corresponding belief entropy, denoted as $E_{di}\left(E_{i}\right)$, i=1,2,3, and the global belief entropy, denoted as $E_{d}^{g}\left(E_{i}\right)$, i=1,2,3, are calculated in Table 5.

With Equation (11), the degree of orness α of the case study, denoted as $\alpha \left(E_{i}\right),$ is calculated as follows:

(26) $$\alpha \left(E_{i}\right)=\frac{1}{2}\left(e^{0.3712\cdot \ln0.5}+0.5^{0.5884}\right)=0.7189.$$

According to the Maximum Entropy Method defined in Equation (10), the weight vector of the evidence, denoted as $W\left(E_{i}\right)={\left(w_{1},w_{2},w_{3}\right)}^{T},$ can be calculated, and the result is

(27) $$W\left(E_{i}\right)={\left(0.5771,0.2836,0.1393\right)}^{T}.$$

The inducing variable, denoted as $S_{i}\left(E_{i}\right)$ (i=1,2,3), can be calculated based on Equation (19) and the parameters in Table 4, and the results are shown as follows:

(28) $$\begin{array}{c} S_{1}\left(E_{1}\right)=1-0.1916=0.8084,\\ S_{2}\left(E_{2}\right)=1-0.3477=0.6523,\\ S_{3}\left(E_{3}\right)=1-0.2033=0.7967. \end{array}$$

Combining the inducing variables with the parameters in Table 5, the OWA pairs $<S_{i}\left(E_{i}\right),\;E_{i}>$, i=1,2,3, are ordered as follows:

(29) $$\begin{array}{c} \langle 0.8084,E_{1}\rangle ,\\ \langle 0.7967,E_{3}\rangle ,\\ \langle 0.6523,E_{2}\rangle . \end{array}$$

Now, the BPAs in Table 3 can be modified according to Equation (8), and the weighted average evidence are as follows:

(30) $$m\left(F_{1}\right)=0.5517,\;\;\;m\left(F_{2}\right)=0.1975,\;\;\;m\left(F_{2},F_{3}\right)=0.0930,\;\;\;m\left(\Theta \right)=0.1577.$$

Finally, combining the weighted average evidence with Dempster’s combination rule by four times, the fusion results are as follows:

(31) $$m\left(F_{1}\right)=0.9123,\;\;\;m\left(F_{2}\right)=0.0810,\;\;\;m\left(F_{2},F_{3}\right)=0.0027,\;\;\;m\left(\Theta \right)=0.0039.$$

The fused results with the proposed method are compared with the method in [66] where this case study comes from, and the comparison result is shown in Table 6.

It can be concluded from Table 6 that the proposed method has the most distinguishable fusion results on sensor reports, which means a clear indicator on the most possible fault type. The highest belief degree on fault type $F_{1}$ is 91.23%, which is higher than the method with Fan et al’s method with more than 10%. This is helpful for decision-making in real applications. While the fusion results of fault type $F_{1}$ and $F_{2}$ with the conventional Dempster’s rule of combination are close to each other, it is hard to judge which fault has occurred. The case study verifies the effectiveness of the proposed method. In addition, the case study indicates a better performance of the proposed method in comparison with some of the existing methods.

### 4.3. Discussion

The effectiveness of the proposed method is verified according to the applications based on both artificial data and the experiment adopted from the literature.

A few reasons contribute to the success of the new method. Firstly, not only the distance of evidence, but also the belief entropy and the IOWA operator are taken into consideration, which means more available information are used while doing information processing. Thus, information loss is decreased. Secondly, the way of getting the degree of orness *a* of IOWA (based on belief entropy and evidence distance) is data-driven, which is more reliable compared with some subjective methods. Finally, the final fused rule is based on Dempster’s rule of combination. The merits of Dempster’s rule of combination, such as satisfying the commutativity and associativity, contribute to the effectiveness of the proposed method.

In the fault diagnosis (FD) research area, an FD technique is good if a new method can guarantee that there is no false alarm, no missed detection and a full detection for all considered faulty scenarios [67,68]. The ongoing work of the proposed method should try to focus on this case. In future work, the following situations should be well addressed:

- FD without fault to be sure that the proposed solution doesn’t give false alarm,
- FD with a misalignment fault to highlight that we detect this fault well,
- FD with pedestal fault.

## 5. Conclusions

In this paper, in the frame of Dempster–Shafer evidence theory, an improved evidential-IOWA sensor data fusion approach is proposed in dealing with a multi-source data-based fault diagnosis problem. Before applying sensor data fusion for final decision-making, the sensor data comes from different independent sources modelled, as BPA is pre-processed to avoid unreasonable fusion results that may be caused by conflicting evidence. In the new method, the IOWA operator is used to determine the weight of different sensor data sources, and the parameter of the IOWA is based on the distance of evidence and the belief entropy. The proposed method has a better performance in conflict management and fault diagnosis due to the fact that the information volume of each piece of evidence is taken into consideration. The proposed method outperforms the other methods according to the applications.

The ongoing work of the proposed method will be focused on some basic rules of fault diagnosis in industrial environmental scenarios, e.g., no missed detection and a full detection for all considered faulty scenarios should be strictly obeyed while applying the fault diagnosis technique.

## Figures and Tables

**Table 1:** The basic probability assignment (BPA) as an example.

$m_{i}$	$\left\{A\right\}$	$\left\{B\right\}$	$\left\{C\right\}$	$\left\{\mathrm{AC}\right\}$
$m_{1}$	0.41	0.29	0.30	0.00
$m_{2}$	0.00	0.90	0.10	0.00
$m_{3}$	0.58	0.07	0.00	0.35
$m_{4}$	0.55	0.10	0.00	0.35
$m_{5}$	0.6	0.10	0.00	0.30

**Table 2:** Comparison of several existing methods.

BPAs	Methods	m(A)	m(B)	m(C)	m(AC)	Faults
$m_{1},m_{2}$	Dempster’s method [25]	0	0.8969	0.1031	0	*B*
	Murphy’s method [54]	0.0964	0.8119	0.0917	0	*B*
	Deng et al.’s method [55]	0.0964	0.8119	0.0917	0	*B*
	The proposed method	0.0964	0.8119	0.0917	0	*B*
$m_{1},m_{2},m_{3}$	Dempster’s method [25]	0	0.6350	0.3650	0	*B*
	Murphy’s method [54]	0.4939	0.4180	0.0792	0.0090	*A*
	Deng et al.’s method [55]	0.4974	0.4054	0.0888	0.0084	*A*
	The proposed method	0.6960	0.1744	0.1253	0.0056	*A*
$m_{1},m_{2},m_{3},m_{4}$	Dempster’s method [25]	0	0.3321	0.6679	0	*C*
	Murphy’s method [54]	0.8362	0.1147	0.0410	0.0081	*A*
	Deng et al.’s method [55]	0.9089	0.0444	0.0379	0.0089	*A*
	The proposed method	0.9683	0.0020	0.0133	0.0163	*A*
$m_{1},m_{2},m_{3},m_{4},m_{5}$	Dempster’s method [25]	0	0.1422	0.8578	0	*C*
	Murphy’s method [54]	0.9620	0.0210	0.0138	0.0032	*A*
	Deng et al.’s method [55]	0.9820	0.0039	0.0107	0.0034	*A*
	The proposed method	0.9914	0.0001	0.0025	0.0061	*A*

**Table 3:** BPAs for fault diagnosis of the case study [66].

Sensor Report	$\left\{F_{1}\right\}$	$\left\{F_{2}\right\}$	$\left\{F_{2},F_{3}\right\}$	Θ
$E_{1}:m_{1}\left(\cdot \right)$	0.60	0.10	0.10	0.20
$E_{2}:m_{2}\left(\cdot \right)$	0.05	0.80	0.05	0.10
$E_{3}:m_{3}\left(\cdot \right)$	0.70	0.10	0.10	0.10

**Table 4:** The average distance and global distance of $E_{i}$ (i=1,2,3).

Evidence Distance-Based Parameter	$\bar{d_{1}}\left(E_{1}\right)$	$\bar{d_{2}}\left(E_{2}\right)$	$\bar{d_{3}}\left(E_{3}\right)$	$d_{g}\left(E_{i}\right)$
**Calculation Result**	0.1916	0.3477	0.2033	0.3712

**Table 5:** The belief entropy and global belief entropy of $E_{i}$ (i=1,2,3).

Belief Entropy-Based Parameter	$E_{d1}\left(E_{1}\right)$	$E_{d2}\left(E_{2}\right)$	$E_{d3}\left(E_{3}\right)$	$E_{d}^{g}\left(E_{i}\right)$
**Calculation Result**	2.2909	1.3819	1.7960	0.5884

**Table 6:** Fusion results with different methods.

Fault Types	$\left\{F_{1}\right\}$	$\left\{F_{2}\right\}$	$\left\{F_{2},F_{3}\right\}$	Θ
**Only Dempster’s Rule of Combination**	0.4519	0.5048	0.0336	0.0096
**Fan et al’s Method** [66]	0.8119	0.1096	0.0526	0.0259
**The Proposed Method**	0.9123	0.0810	0.0027	0.0039
